# Characterization of the RAS/RAF/ERK Signal Cascade as a Novel Regulating Factor in Alpha-Amanitin-Induced Cytotoxicity in Huh-7 Cells

**DOI:** 10.3390/ijms232012294

**Published:** 2022-10-14

**Authors:** Doeun Kim, Min Seo Lee, Eunji Sung, Sangkyu Lee, Hye Suk Lee

**Affiliations:** 1 Research Institute of Pharmaceutical Sciences, Kyungpook National University, Daegu 41566, Korea; 2 BK21 FOUR-Sponsored Advanced Program for SmartPharma Leaders, College of Pharmacy, The Catholic University of Korea, Bucheon 14662, Korea; 3 BK21 FOUR Community-Based Intelligent Novel Drug Discovery Education Unit, College of Pharmacy, Kyungpook National University, Daegu 41566, Korea

**Keywords:** alpha-amanitin, toxic mushroom, acute liver failure, global phosphoproteome, RAS/RAF/ERK signaling pathway

## Abstract

The well-known hepatotoxicity mechanism resulting from alpha-amanitin (α-AMA) exposure arises from RNA polymerase II (RNAP II) inhibition. RNAP Ⅱ inhibition occurs through the dysregulation of mRNA synthesis. However, the signaling pathways in hepatocytes that arise from α-AMA have not yet been fully elucidated. Here, we identified that the RAS/RAF/ERK signaling pathway was activated through quantitative phosphoproteomic and molecular biological analyses in Huh-7 cells. Bioinformatics analysis showed that α-AMA exposure increased protein phosphorylation in a time-dependent α-AMA exposure. In addition, phosphorylation increased not only the components of the ERK signaling pathway but also U2AF65 and SPF45, known splicing factors. Therefore, we propose a novel mechanism of α-AMA as follows. The RAS/RAF/ERK signaling pathway involved in aberrant splicing events is activated by α-AMA exposure followed by aberrant splicing events leading to cell death in Huh-7 cells.

## 1. Introduction

For several decades, mushrooms have become popular with people interested in well-being [1]. There is a growing interest in harvesting wild edible mushrooms, and ingesting food from nature is becoming more common [2], increasing the possibility of toxic mushroom exposure [3]. Despite these risks, people may confuse edible and toxic mushrooms because of misidentification based on morphology. Toxic mushrooms are classified by their toxic components, such as cyclopeptides, gyromitrin, muscarine, coprine, isoxazoles, orellanine, psilocybin, and gastrointestinal irritants [4]. Poisonous mushrooms containing cyclopeptide toxins are responsible for 90–95% of all deaths resulting from their consumption.

*Amanita phalloides* has the highest rate of fatalities due to intoxication [5,6,7,8]. Amatoxin poisoning has a poor prognosis because of the high risk of liver failure. Although there are no universal treatment guidelines for amatoxin intoxication, supportive care and antidotes are frequently used [9,10,11]. Alpha-amanitin (α-AMA) poisoning is characterized by the accumulation of α-AMA in the liver and kidneys, with no symptoms until extensive damage has occurred [12]. Clinical symptoms of amatoxin ingestion are expected to manifest after several hours (6–24 h) or even days and include nausea, vomiting, diarrhea, abdominal pain, and hematuria [13]. During this period, fever, tachycardia, and metabolic disorders such as hypoglycemia, dehydration, and electrolyte imbalance may occur [14]. Several mechanisms of toxicity have been attributed to amatoxins, associated with their ability to non-covalently bind and inhibit RNA polymerase II (RNAP II) activity within the nucleus [15]. Several experimental studies have examined interactions between amatoxins and RNAP II [16,17].

Protein phosphorylation is a reversibly regulated representative protein modification by kinases and phosphatases. Competition between kinases and phosphatases generates protein phosphorylation, indicating a signaling pathway critical for numerous cellular functions such as proliferation, survival, differentiation, function, and motility [18,19]. Therefore, abnormal regulation of signaling pathways by hyper- or hypophosphorylation could be the causative mechanism of the toxic response. For example, phosphorylation by an extracellular signal-related kinase (ERK) and AMP-activated protein kinase (AMPK) is related to pathological processes that occur after exposure to harmful metals such as cadmium and selenium [20,21]. In addition, toxicity leading to cell death has been reported for various chemicals via p38 mitogen-activated protein kinase (MAPK), protein kinase B (AKT), and protein phosphatase 2A (PP2A) [22,23,24].

It is difficult to identify an unknown signaling pathway that causes the toxicity of a specific toxic compound. However, it can be solved by applying a global quantitative phosphoproteome. In this study, to understand the initial signaling process leading to acute liver failure (ALF) after α-AMA exposure, we profiled the level of protein phosphorylation over 0–12 h after α-AMA treatment of Huh-7 human hepatoma cells. In addition, comparative phosphoproteomics analysis was performed using tandem mass tag (TMT) labeling coupled with titanium dioxide (TiO_2_)-affinity chromatography. The data have shown that activation of the RAS/RAF/ERK signaling pathway and hyperphosphorylation of spliceosomal proteins are key mechanisms of α-AMA-induced hepatotoxicity. Moreover, inhibition of induced ERK activity through chemical ERK inhibitors suggests the possibility of controlling hepatotoxicity induced by α-AMA.

## 2. Results

### 2.1. Characterization of Cytotoxicity Induced by α-AMA in Human Hepatoma Cells

Toxic concentrations of α-AMA were evaluated in human Huh-7 cells to explore the protein phosphorylation involved in early toxicity following α-AMA exposure in the human liver. α-AMA was added to Huh-7 cells for 24 h at a concentration of 0.5 to 10 μM, and cell viability was evaluated using the CCK-8 assay (Figure S1A). Doxorubicin (DOX) treatment was used as a control for cell viability. Although a previous study reported that 2 μM α-AMA produced reversible hepatic damage in a human normal liver cell line, we determined 5 μM α-AMA as the concentration to induce cytotoxicity in Huh-7 cells [25]. In this study, treatment with 5 μM α-AMA resulted in cell death of approximately 10% of the total cells by CCK-8 assay and induced morphological differences (Figure S1B). The 5 μM α-AMA was considered to be the initial toxicity condition chosen as an early stage of liver failure to look for changes in the proteome, consistent with our previous study results [26].

### 2.2. Time-Dependent Quantitative Protein Phosphorylation Analysis by α-AMA Treatment

Protein phosphorylation was evaluated by Western blotting using pan-specific Ser-, Thr-, and Tyr-phosphorylation antibodies to determine the dynamics of protein phosphorylation after α-AMA treatment (Figure S2). Significant protein degradation was not observed on the SDS-PAGE. Instead, phosphorylated proteins were observed to increase slowly in a time-dependent manner.

**Figure 1:**
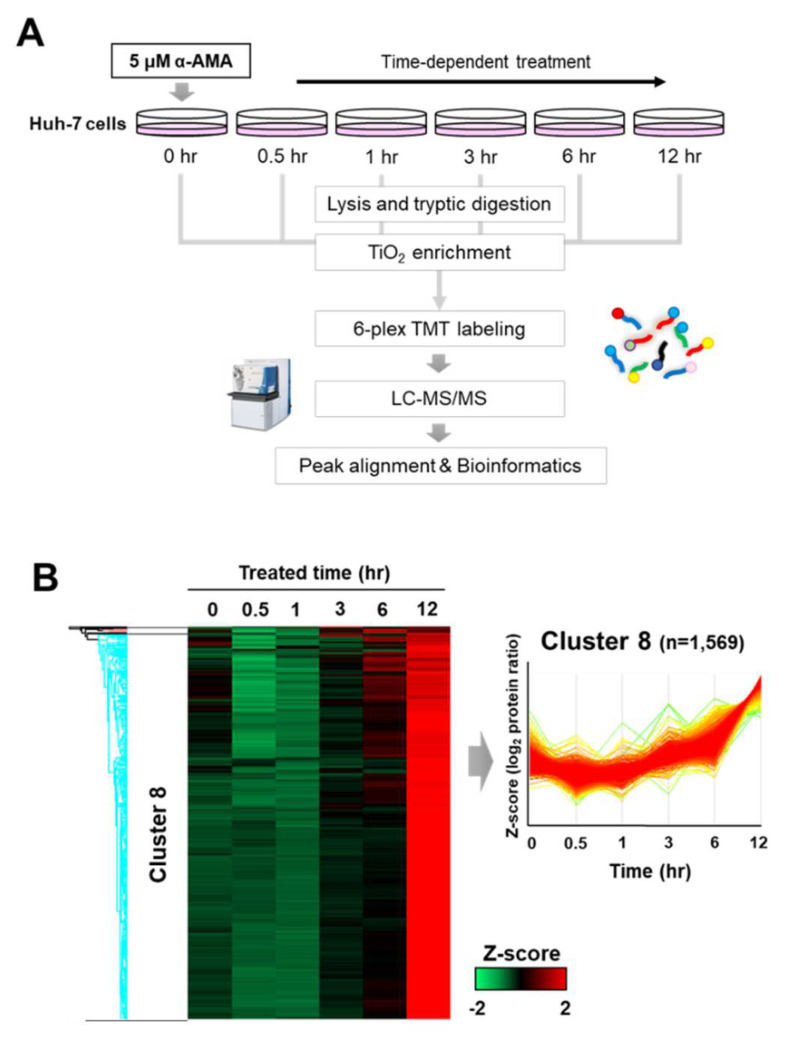
**Systematic time profiling of the global phosphoproteome after α-amanitin (α-AMA) treatment of Huh-7 cells**. (**A**) Schematic workflow of comparative phosphoproteome after treatment with α-AMA (5 μM) for 12 h. (**B**) Time-dependent unsupervised hierarchical clusters of phosphorylation after treating Huh-7 cells with α-AMA. Heatmap between each time point for the α-AMA treatment group after Z-score normalization.

To identify the dynamics of protein phosphorylation by α-AMA treatment in Huh-7 cells, a comparative phosphoproteome analysis was performed (Figure 1A). To enrich global phosphopeptides at each period, TiO_2_-affinity chromatography was first performed, and labeling was performed with 6-plex TMT for relative quantification at each period. The samples were analyzed by nano-flow LC-MS/MS with technical duplicates. Overall, we identified 2785 phosphopeptides (1598 phosphoproteins) and quantified 1598 phosphopeptides (763 phosphoproteins) (localization probability > 0.75 and FDR < 1%) (Table S1). The ratio of each group was calculated using the ratio of the reporter ion intensity of the α-AMA samples after and before treatment (0 h-treated samples).

The tendency of protein phosphorylation level changes was divided into eight clusters according to the unsupervised hierarchical Z-score clustering (Figure 1B). The level of phosphorylation in Cluster 8 increased over time, consistent with the results of Western blotting showing increased phosphorylation following α-AMA treatment. Most of the quantified phosphorylation (1569 phosphopeptides) belonged to Cluster 8.

### 2.3. Characterization of RAS/RAF/ERK Signal Cascade Related to α-AMA-Induced Hepatotoxicity

**Figure 2:**
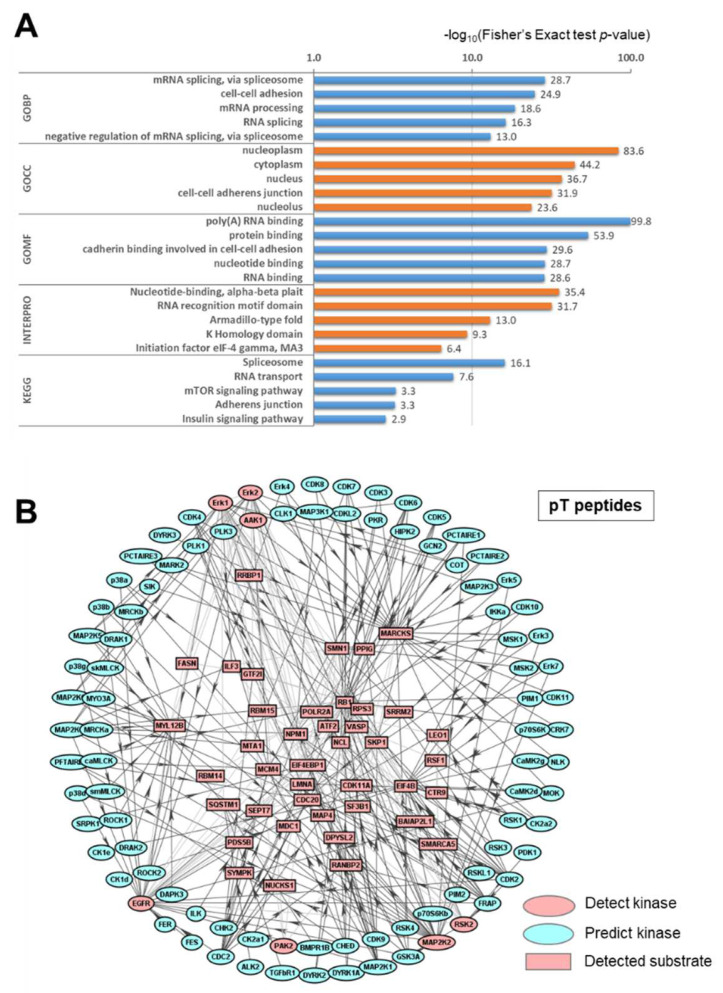
**Characterization of time-dependently upregulated phosphorylation by α-amanitin (α-AMA) treatment of Huh-7 cells.** (**A**) DAVID-generated Gene Ontology (GO) enrichment and Kyoto Encyclopedia of Genes and Genomes (KEGG) pathway analysis of Cluster 8. (**B**) Kinase–substrate interaction analysis of phospho-threonine proteins using iGPS 1.0. Circle (Kinase), Square (Substrate), Pink (Detected), and Blue (Predicted).

We characterized the phosphorylation in Cluster 8 using GO, Interpro, and KEGG enrichment analyses using the DAVID bioinformatics resources tool to document the time-dependent phosphorylation increase after 5 μM α-AMA treatment (Figure 2A). In the GO biological process (GOBP) categories, mRNA splicing, cell–cell adhesion, and mRNA processing were in Cluster 8. The GO cell component (GOCC) categories showed that upregulated phosphorylation was present in the nucleoplasm, cytoplasm, nucleus, cell–cell adherens junction, and nucleolus. Time-dependent increased protein phosphorylation was also observed in GO molecular function (GOMF) categories, such as poly(A) RNA binding, protein binding, cadherin binding involved in cell-cell adhesion, nucleotide binding, and RNA binding. The Interpro identified nucleotide-binding, RNA recognition motif domain, armadillo-type fold, K homology domain, and initiation factor eIF-4 gamma. Finally, KEGG analysis revealed that gradually increasing phosphoproteins were involved in the spliceosome, RNA transport, mTOR signaling pathway, adherens junction, and insulin signaling pathway. In particular, the spliceosome category included 73 phosphorylations in 32 proteins, including splicing factor 45 (RNA binding motif protein 17 (RBM17), SPF45) and splicing factor U2AF 65 kDa subunit (U2AF2, U2AF65) that modulate factors of RAS/RAF/ERK signaling [27,28]. The mTOR signaling pathway category included 17 phosphorylations in nine proteins, including two kinases, mitogen-activated protein kinase 1/2 (extracellular signal-regulated kinase 2 (ERK2), MAPK 1/2), and non-specific serine/threonine protein kinase.

In addition, to discover the kinases that play a key role in time-dependently upregulated phosphorylation in Cluster 8, we sorted phosphopeptide sequences by phospho-serine and phospho-threonine and input the sequences to iGPS 1.0 to find a kinase and substrate protein network (Figure 2B and Figure S4) [29]. In the phospho-threonine peptide group, 85 kinases were predicted to interact with the identified phosphosites; seven kinases were detected in Cluster 8. The kinases are extracellular signal-regulated kinase 1 and 2 (ERK1/2), AP2-associated protein kinase 1 (AAK1), receptor protein-tyrosine kinase (EGFR), serine/threonine-protein kinase PAK 2 (PAK2), dual-specificity mitogen-activated protein kinase kinase 2 (MAP2K2), and non-specific serine/threonine protein kinase (RSK2, RPS6KA3). Taken together with the results of the DAVID and kinase–substrate interaction analysis, we found that the RAS/RAF/ERK signaling cascade was involved when initial hepatotoxicity was induced after exposure to α-AMA [30].

### 2.4. Investigation of RAS/RAF/ERK Signal Pathway Role for α-AMA-Induced Hepatotoxicity

**Figure 3:**
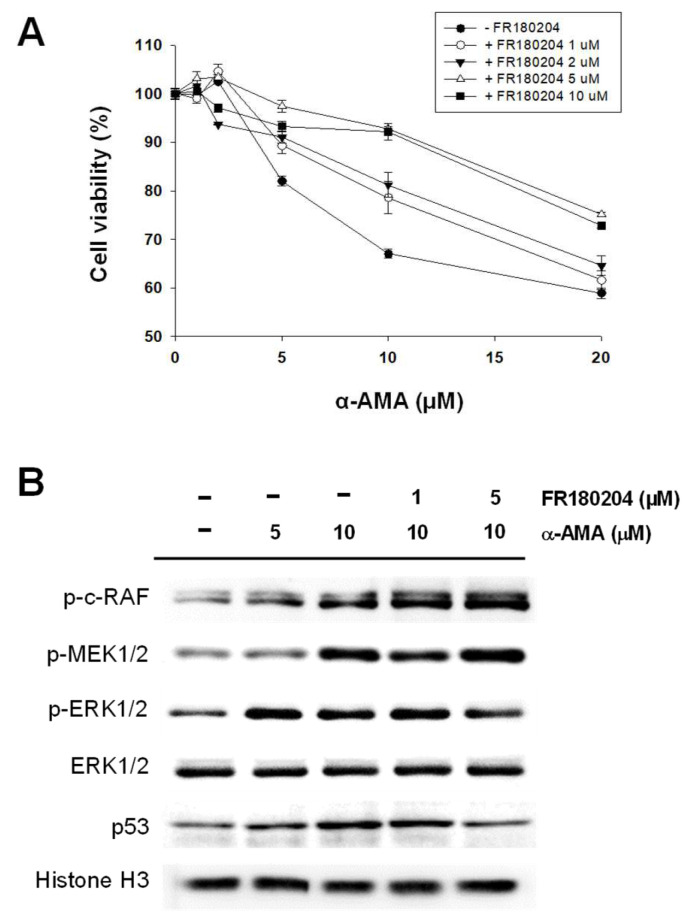
**Identification of RAS/RAF/ERK signaling pathway related to the toxicity of α-amanitin (α-AMA) in Huh-7 cells.** (**A**) Cell viability assay for α-AMA treatment with ERK1/2 inhibitor. Cell density was 5 × 10^3^ cells/well in a 96-well plate. The viability of Huh-7 cells was detected by CCK-8 reagent after α-AMA and ERK1/2 inhibitor treatment for 24 h. The data are presented as the means ± SEM (*n* = 3). (**B**) Immunoblotting assay of RAS/RAF/ERK cascade after α-AMA and ERK1/2 inhibitor (FR180204) treatment of Huh-7 cells.

To check the role of RAS/RAF/ERK signal cascade, we treated Huh-7 cells for 24 h with α-AMA (0–20 μM) and with 1, 2, 5, and 10 μM ERK1/2 inhibitor (FR180204) (Figure 3A) [31,32]. The linear plot for the cell viability assay showed that cell viability was gradually reduced by α-AMA in a concentration-dependent manner. However, it recovered with increasing ERK1/2 inhibitor concentration. Moreover, we found that p53 levels were increased according to α-AMA concentration; however, p53 levels were gradually reduced after treatment with the ERK1/2 inhibitor at 10 μM α-AMA (Figure 3B).

We verified the correlation between the RAS/RAF/ERK signaling cascade and α-AMA-induced cytotoxicity using Western blotting (Figure 3B). In the RAS/RAF/ERK signaling cascade, such as p-c-RAF and p-MEK1/2, factors upstream of ERK were increased by treatment with α-AMA but were not affected by ERK inhibitor treatment in Huh-7 cells. However, while the level of ERK did not change, the increase in p-ERK expression by α-AMA was slightly decreased by ERK1/2 inhibitor treatment. It was established that activation of the RAS/RAF/ERK signaling cascade by α-AMA can cause hepatotoxicity that could be alleviated by selective inhibitors.

**Figure 4:**
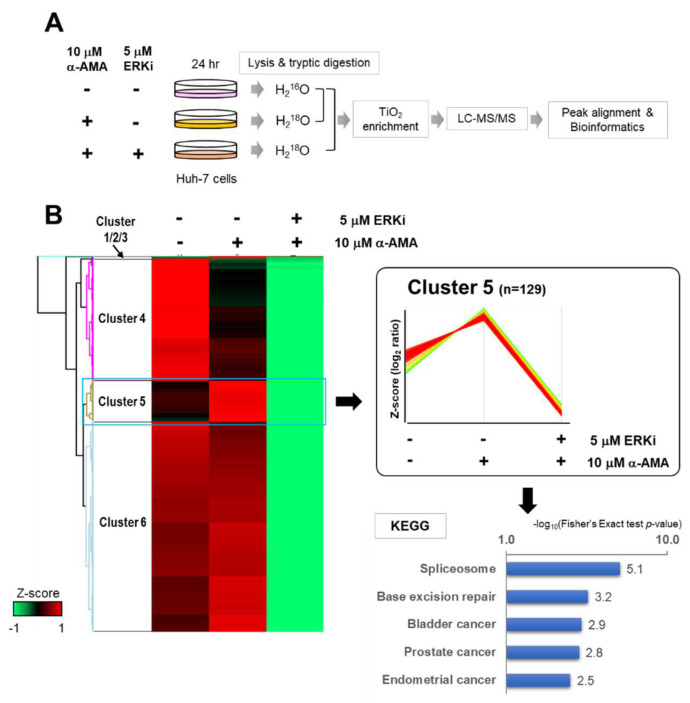
**Global phosphoproteomic profiling of α-amanitin (α-AMA) target factors induced by RAS/RAF/ERK signal-pathway activation**. (**A**) Flow chart of the comparative phosphoproteome. Huh-7 cells were treated with α-AMA (10 μM) and/or ERK1/2 inhibitor (FR180204, 5 μM) for 24 h. (**B**) Cluster 5 selected by unsupervised hierarchical clusters of phosphorylation after α-AMA treatment of Huh-7 cells with ERK 1/2 inhibitor. Heatmap between each time point of α-AMA treatment after Z-score normalization. DAVID-generated Kyoto Encyclopedia of Genes and Genomes (KEGG) pathway analysis of Cluster 5.

To identify downstream factors affecting the RAS/RAF/ERK signaling cascade of toxicity induced by α-AMA, we carried out a comparative phosphoproteome analysis of an ERK1/2 inhibitor treatment of Huh-7 cells (Figure 4A). To quantify the phosphopeptides in each group, we applied ^16/18^O-labeling during trypsin digestion and enrichment by TiO_2_-affinity chromatography. The phosphorylation data for each group are shown in Table S3. We quantified 1203 phosphopeptides in 2200 identified phosphopeptides in the mixed control and 10 μM α-AMA-treated groups. Only 449 phosphopeptides out of 764 identified phosphopeptides were quantified in the mixed group of control and 10 μM α-AMA-treated group with 5 μM ERK 1/2 inhibitor. The decreased phosphorylation in the ERK 1/2 inhibitor-treated group was a result of the inhibition of the RAS/RAF/ERK signaling cascade.

**Figure 5:**
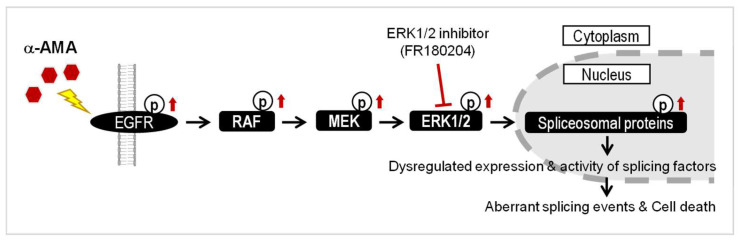
**Activation of RAS/RAF/ERK signaling cascades related to *α*-amanitin (*α*-AMA)-induced cytotoxicity in Huh-7 cells.** EGFR, epidermal growth factor receptor; RAF, RAF proto-oncogene serine/threonine-protein kinase; MEK, dual specificity mitogen-activated protein kinase; ERK1/2, extracellular signal-regulated kinase 1/2; SAM68, Src associated in mitosis 68 kDa protein; U2AF65, splicing factor U2AF 65 kDa subunit; SPF45, splicing factor 45. Arrow means the increase.

Therefore, when analyzing the changes in protein phosphorylation levels by unsupervised hierarchical clustering, we paid attention to Cluster 5, which increased by α-AMA treatment and decreased sequentially by the ERK 1/2 inhibitor (Figure 4B). In Cluster 5, 129 phosphorylations were classified, and the spliceosome was included in the KEGG category through DAVID analysis. The seven proteins were included in the spliceosome class, and two phosphosites were in U4/U6. U5 tri-snRNP-associated protein 2 (Ser82) and SPF45 (Ser155) were detected at the same sites in Cluster 8 after only α-AMA treatment (Table S4). Although it is involved in various functions in the RAS/RAF/ERK signaling cascade, it is related to the increased phosphorylation of proteins in the spliceosome about α-AMA toxicity (Figure 5).

## 3. Discussion

In this study, to identify the initial signaling pathway involved in hepatotoxicity that occurs following α-AMA exposure, global protein phosphorylation changes up to 12 h after treatment with 5 μM α-AMA in Huh-7 cells, a human hepatoma cell line, were profiled based on comparative phosphoproteomic analysis. Most of the detected protein phosphorylation was increased 12 h after α-AMA, and it was found by unsupervised hierarchical clustering using a Z-score analysis that the proteins with increased phosphorylation belonged to Cluster 8. DAVID analysis of the proteins included in Cluster 8 showed that phosphorylation of proteins related to the spliceosome was increased (Figure 2A). In addition, the RAS/RAF/ERK signaling cascade was included in Cluster 8 from the analysis of the kinase–substrate interaction based on iGPS 1.0 (Figure 2B).

To establish a cytotoxicity model by α-AMA in this study, we treated Huh-7 cells with 5 μM of α-AMA, which is higher than the concentration that induces ALF to which patients are exposed in actual clinical practice. Various concentrations have been reported from previous studies, it is measured in patient plasma with acute accidental poisoning with wild mushrooms at the maximum ng/mL level [33,34]. It is ideal to determine the concentration to be treated in the cell line by reflecting the clinical concentration; however, the assessed concentration may not match due to the heterogeneity between the two systems. Although the concentration of α-AMA treated in Huh-7 cells is higher than that detected in patient blood, it is the concentration at which irreversible damage occurs in hepatocytes [25], which may reflect the initial intracellular damage caused by α-AMA.

The RAS/RAF/ERK signaling cascade was a key factor in cytotoxicity induced by α-AMA in Huh-7 cells. Upon treatment with α-AMA, phosphorylation of RAF and MEK upstream of ERK1/2 increased, leading to the activation of ERK1/2 (Figure 3B) [30]. However, when ERK activity induced by α-AMA in Huh-7 cells was inhibited by treatment with an ERK1/2 inhibitor, the cytotoxicity caused by α-AMA was reduced (Figure 5). In many previous studies, the RAS/RAF/ERK signaling cascade was reported to be associated with cell proliferation, differentiation, migration, senescence, and apoptosis [21,35]. Additionally, as an unexpected role of xenobiotic exposure, the activation of the RAS/RAF/ERK signaling cascade is the cause of toxicity.

Heavy metals such as lead, chromium, arsenic, mercury, nickel, and cadmium cause hepatotoxicity by generating ROS that cause numerous injuries and undesirable changes in the liver [36]. ROS upregulate ERK1/2, causing an abnormal mitochondrial division and eventually inducing cell death [37,38]. The RAS/RAF/ERK signaling cascade mediates cellular responses to diverse environmental toxicants, including heavy metals, and may trigger CNS disorders via modulation of the MAPK pathways [39]. Chlorpyrifos, an organophosphate, induces cytotoxicity and neuronal death by increasing p-p38 and p-ERK expression and caspase-3 levels [40]. Perfluorooctane sulfonic acid (PFOA), a persistent organic pollutant, increases TNF-α and IL-6 expression, partly by increasing ERK1/2-MAPK/NF-κB [41]. As a result, since activation of the RAS/RAF/ERK signaling pathway by toxicant exposure can cause toxicity, it is supported that the activation of RAS/RAF/ERK signaling by α-AMA can be a mechanism causing cytotoxicity in Huh-7 cells.

Furthermore, increased phosphorylation of splicing factors, such as SRSFs and RNA-binding proteins, was investigated in Huh-7 cells after α-AMA treatment (Table S2). The phosphorylation level of proteins involved in the splicing process is very important for pre-mRNA splicing, regulated by various signaling pathways, including the RAS/RAF/ERK cascade [28]. One of the target factors of the RAS/RAF/ERK signaling pathway is SAM68, a prototype regulator of alternative splicing [42]. SAM68 interacts with the splicing factor U2AF65, and phosphorylation by ERKs reduces the affinity of the SAM68/U2AF65 complex to *CD44* pre-mRNA [43]. Although phosphorylation of SAM68 was not detected in this study, we identified an increase in U2AF65 phosphorylation (S475) following activation of the RAS/RAF/ERK signaling pathway by α-AMA treatment in Huh-7 cells. Another target factor of the RAS/RAF/ERK signaling pathway is SPF45, which is related to regulating alternative mRNA splicing factors [44]. ERK2 phosphorylates SPF45 on Thr71 and Ser222, whereas the phosphorylation on Ser155 was observed in our study [44]. Although the effect of Ser155 phosphorylation on the function of SPF45 should be further studied, we found that Ser155 was increased by α-AMA and decreased by the ERK 1/2 inhibitor (Table S4). This result also supports that the RAS/RAF/ERK signaling pathway is involved in α-AMA toxicity.

In conclusion, based on a comparative phosphoproteome approach, we suggest that activation of the RAS/RAF/ERK signaling cascade is a new mechanism involved in cytotoxicity caused by exposure to α-AMA in Huh-7 cells. Further, the toxicity could be controlled with an ERK 1/2 inhibitor; validation in animal experiments should be conducted in the future. If confirmed, the utility of the ERK 1/2 inhibitor as a therapeutic target could clinically reduce the high risk of liver failure caused by ingestion of amatoxin.

## 4. Materials and Methods

### 4.1. Cell Culture

Human hepatocyte-derived carcinoma cells (Huh-7) were maintained at 37 °C in 5% CO_2_ in Dulbecco’s Modified Eagle Medium (Hyclone Laboratories Inc., Logan, UT, USA) supplemented with 10% fetal bovine serum (Hyclone Laboratories Inc.) and 1 × penicillin-streptomycin (Gibco^TM^, Grand Island, NY, USA). All experiments were performed using Huh-7 cells under passage 30.

### 4.2. Cell Cytotoxicity Check through CCK-8 Assay

To determine the inhibitory concentration of α-AMA before comparative proteomic analysis in Huh-7 cells, the cytotoxicity of α-AMA was evaluated using a CCK-8 reagent (Dojindo Molecular Technologies, Kumamoto, Japan). CCK-8 assay is a sensitive colorimetric method used to evaluate cell viability in the context of proliferation and death. In cells, dehydrogenases produce a formazan dye in proportion to the number of living cells. Huh-7 cells were cultured in Dulbecco’s Modified Eagle Medium (Hyclone Laboratories Inc.) supplemented with 10% fetal bovine serum (Hyclone Laboratories Inc.) and 1 × penicillin-streptomycin (Gibco) at a concentration of 5 × 10^3^ cells/well in 96-well plates, and incubated for 18 h. Next, the cells were washed with 1 × phosphate-buffered saline (Gibco). The cell medium was then replaced with fresh cell media containing 1 × penicillin-streptomycin and α-AMA at 2, 5 and 10 μM concentrations and incubated for 24 h. DOX (Sigma-Aldrich, St. Louis, MO, USA) was used as a positive control. Finally, the cell medium was removed, and fresh cell media mixed with the cytotoxicity-checking CCK-8 reagent was added. The absorbance was measured at 450 nm using a spectrophotometer.

To check the effect of the ERK inhibitor (FR180204; Sigma-Aldrich), Huh-7 cells were grown in Dulbecco’s Modified Eagle Medium (Hyclone Laboratories Inc.) supplemented with 10% fetal bovine serum (Hyclone Laboratories Inc.) and 1 × penicillin-streptomycin (Gibco) at a concentration of 5 × 10^3^ cells/well in 96-well plates and incubated for 18 h. The cells were then washed with 1 × phosphate-buffered saline (Gibco). Cells were then transferred to new cell media containing 1 × penicillin-streptomycin and α-AMA at concentrations of 1, 2, 5, 10 and 20 μM. Then, 1, 2, 5 and 10 μM ERK1/2 inhibitors were added to each α-AMA concentration group, and cells were incubated for 24 h.

### 4.3. Preparation of Proteins from Hepatocytes and Trypsin Digestion

α-AMA-treated Huh-7 cells were harvested and directly added to 500 μL of 8 M Urea (Sigma-Aldrich) in 100 mM Tris (VWR International, Radnor, PA, USA) containing protease and phosphatase inhibitors (Thermo Fisher Scientific, Waltham, MA, USA). The collected cells for one minute (output 30%, 5-s on and off intervals) and then centrifuged at RT at 16,000× *g* for 10 min to separate the soluble proteins from the cell debris. The supernatant was collected from the top fraction and placed in new sample tubes, and the protein concentration was determined using a BCA kit (Thermo Fisher Scientific). Duplicate samples were harvested from each treatment group, and proteins were extracted. Protein samples (100 μg) were placed in new sample tubes, and 5 mM dithiothreitol (Sigma-Aldrich) was added for cysteine residue reduction at 56 °C for 30 min. The samples were then treated with 15 mM iodoacetamide (Sigma-Aldrich) in the dark for 30 min to alkylate cysteine groups. Next, samples were diluted two-fold for trypsin digestion. Following a pH check, trypsin (2 μg) was directly added to the samples and allowed to digest for 18 h at 37 °C. Then, 1% trifluoroacetic acid (Sigma-Aldrich) was added to complete the digestion step. The peptides were dried in a speed-vac dryer at a low temperature.

### 4.4. Sample Preparation for Comparative Phosphoproteomics Analysis

The phosphopeptides were enriched using TiO_2_ Phosphopeptide Enrichment Tips (Thermo Fisher Scientific). The TiO_2_ tip was activated in 20 μL buffer A (40% ACN with 4% TFA) and equilibrated in 20 μL buffer B (buffer A with 25% lactic acid). The peptides were dissolved in 150 μL of buffer B using a sonicator and loaded onto the TiO_2_ tip. Phosphopeptides were washed twice with buffer B and three times with buffer A and eluted using 50 μL of 1.5% ammonium hydroxide solution and 50 μL of 5% pyrrolidine. The sample was desalted following the manufacturer’s instructions using GL-Tip™SDB and GL-Tip™GC (GL Science Inc., Tokyo, Japan).

Dried peptide samples were dissolved in 50 mM tetraethylammonium bromide (Sigma-Aldrich) for 6-plex TMT reagent labeling (Thermo Fisher Scientific). After checking the peptide concentration using a Pierce™ quantitative colorimetric peptide assay kit (Thermo Fisher Scientific), equal amounts of peptides from each group were labeled and placed in a sample tube. Pooled peptide samples were fractionated using a Pierce™ High pH Reversed-Phase Peptide Fractionation Kit (Thermo Fisher Scientific).

We harvested 10 μM α-AMA-treated Huh-7 cells with and without 5 μM ERK1/2 inhibitor and added to 500 μL of 8 M Urea (Sigma-Aldrich) in 100 mM Tris (VWR International) containing protease and phosphatase inhibitors (Thermo Fisher Scientific) to investigate the effect of the ERK1/2 inhibitor. The trypsin-digested peptides were collected as described above. The α-AMA and ERK1/2 inhibitor treatment groups were labeled with ^18^O water (Cambridge Isotope Laboratories, Inc., Cambridge, MA, USA) for quantitative analysis based on the ^18^O/^16^O ratio. Peptide concentrations were determined using a Pierce™ quantitative colorimetric peptide assay kit (Thermo Fisher Scientific). Equal amounts of peptides from each group were blended 1:1 with the control group. Phosphopeptide enrichment was performed as previously described.

### 4.5. Instruments

All samples were dissolved in 10 μL of solution A (2% acetonitrile in 0.1% formic acid), and 2 μg of each fraction was loaded onto an Ultimate 3000 RSLCnano system connected to a PepMap™ RSLC C_18_ analytical column and Acclaim PepMap™ 100 trap column. Samples were eluted using the gradient liquid chromatography method (5–30% acetonitrile for 150 min) and analyzed using an LTQ-Orbitrap Velos mass spectrometer in positive ion mode at the Mass Spectrometry Convergence Research Center. Quantitative mass spectrometry analyses were performed in duplicate for each pooled peptide sample. The electrospray voltage was set to 2.0 kV for the TMT-labeled sample analysis. The precursor ion scans were acquired at a resolution of 60000. The automatic gain control (AGC) target value for the MS scan of 1.0 × 10^6^ higher-energy collisional dissociation collision (HCD) mode was used to obtain MS_2_ (R = 7500). The data-dependent mode producing ten of the most abundant ions from the full scans was fragmented in the HCD mode with 40% normalized collision energy (NCE). For ^18^O-labeled sample analysis, the MS_2_ parameter changed from the HCD mode to the collision-induced dissociation (CID) mode with 30% NCE. The other parameters were the same as those used in the TMT-labeled sample analysis.

### 4.6. Phosphoproteome Data Analysis and Bioinformatics Analysis

All mass spectra data were input into MaxQuant 1.5.1.0 [45], and the human proteome database (updated 13 December 2018) was downloaded from Uniprot to obtain bioinformatics information. Protein and peptides were obtained using the following parameters: trypsin/P for cleavage enzyme permitting up to 2 missed cleavages; 10 ppm for precursor ions and 0.02 Da for fragment ions of mass error; carbamidomethylation on Cys for fixed modification and oxidation on Met, phosphorylation on Ser, Thr and Tyr, and acetylation on the protein N-terminus for variable modifications. The false discovery rate (FDR) for proteins, peptides, and phosphosites was set to 1%. The minimum length of the peptide was set to 7. The site localization probability was set to >0.75 for selected specific phosphorylation sites. All other MaxQuant parameters were set to their default values. Data are available in ProteomeXchange with the identifier PXD035817 for α-AMA and PXD035758 for α-AMA+ERK1/2 inhibitor.

The DAVID Functional Annotation Bioinformatics Microarray Analysis web-based software was used for Gene Ontology (GO), InterPro, and KEGG pathway analyses [46]. Perseus 1.6.0.7, depending on the phosphoprotein regulation patterns, was used for unsupervised hierarchical clustering [45]. All reported ion intensities from the treatment groups were divided by the reporter ion intensity from the control group, and these data were normalized using Z-score normalization to categorize proteins based on abundance-changing tendencies. Hierarchical clustering based on Euclidean distance was applied to cluster the normalized scores, and average linkage clustering was used to process the k-means clustering. Heat map clustering was used to visualize the data.

The STRING analytical tool was used to profile the protein–protein networks. iGPS 1.0 was used to identify kinase-specific p-sites and systematically elucidate site-specific kinase-substrate relationships [28]. The STRING analytical tool (https://string-db.org/) was used to search for specific protein networks [47].

### 4.7. Phosphoprotein Screening by Immunoblotting

Proteins (10 μg) were separated by SDS-PAGE using 12% tris-glycine polyacrylamide gel electrophoresis and then transferred to a PVDF membrane using a wet blotting system (Roche, Basel, Switzerland) to profile phosphoproteins in hepatocytes. Membranes were blocked with 5% BSA in TBST (20 mM tris, 500 mM sodium chloride, 0.1% Tween-20, pH 7.5) for 4 h at room temperature (RT) and then incubated with primary antibodies at 4 °C for 18 h. The membranes were washed thrice with TBST for 10 min and then incubated with secondary antibodies for 1 h at RT. Signals were detected using iBright 1500 (Thermo Fisher Scientific) and ECL Prime Immunoblotting Detection Reagent (Cytiva, Marlborough, MA, USA).

To verify the phosphoproteomics results, primary antibodies specific to p53 (Cell Signaling Technology, Danvers, MA, USA; P/N 2524S), histone H3 (Cell Signaling Technology; P/N 9715S), α-tubulin (Abcam, UK; P/N ab52866), phospho-serine (Abcam, P/N ab9332), phospho-threonine (Cell Signaling Technology, P/N 9381S), phospho-tyrosine (Cell Signaling Technology, P/N 9411S), phospho-c-Raf (Cell Signaling Technology, P/N 9421S), phospho-MEK1/2 (Cell Signaling Technology, P/N 9154S), phospho-ERK1/2 (Cell Signaling Technology, P/N 9101S), and ERK1/2 (Cell Signaling Technology, P/N 9102S) were used.

## Data Availability

Data are available via ProteomeXchange with identifier PXD035817 for α-AMA and PXD035758 for α-AMA+ERK1/2 inhibitor.

## Supplementary Materials

The following supporting information can be downloaded at: https://www.mdpi.com/article/10.3390/ijms232012294/s1.
