# Supplementary material for: Characterization of the RAS/RAF/ERK Signal Cascade as a Novel Regulating Factor in Alpha-Amanitin-Induced Cytotoxicity in Huh-7 Cells
Source: Int J Mol Sci. 2022 Oct 14;23(20):12294. doi: 10.3390/ijms232012294 (PMC9603094; doi:10.3390/ijms232012294)
Supplement: Supplementary file 1 [file ijms-23-12294-s001.zip › 20221013_aAMA_supplement infom_final.pdf]

Supplemental information

Characterization of RAS/RAF/ERK Signal Cascade as a Novel Regulating Factor in Alpha–Amanitin Induced Cytotoxicity in Huh-7 cells

Doeun Kim [1], Min Seo Lee [2], Eunji Sung [1], Sangkyu Lee [1,3,*] and Hye Suk Lee [2,*]

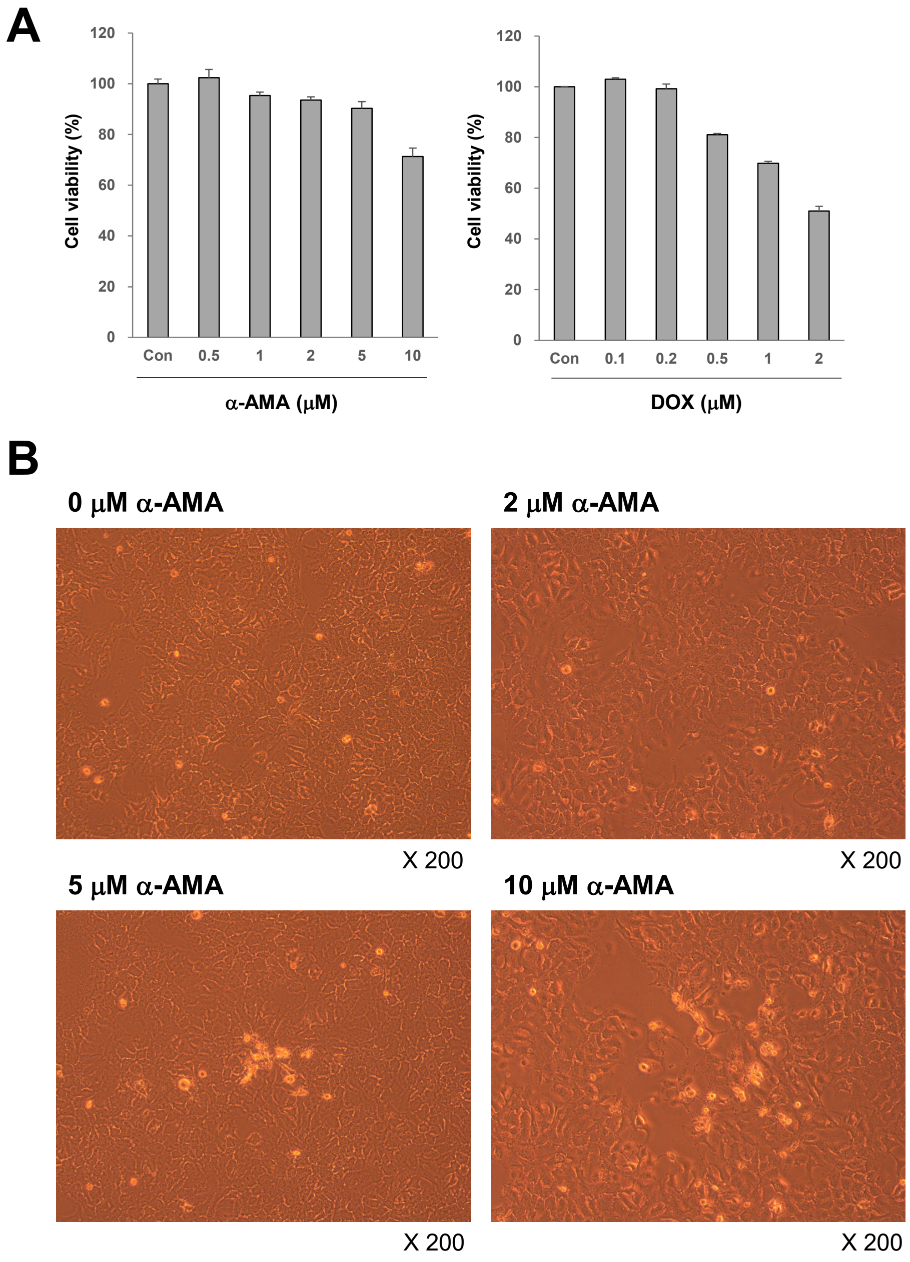

Figure S1. **Alpha–amanitin (α–AMA) induced cytotoxicity in Huh–7 cells. A.** Cell viability assay after α–AMA treatment. Cell density was 5 × 10^3 cells/well in 96–well plate.

The CCK−8 assay kit was used for checking cell viability. Doxorubicin (DOX) was treated as an experiment control. Data are showed by the mean ± SD. **B.** Cell morphology changes. Microscopical magnification was set to 200 X.

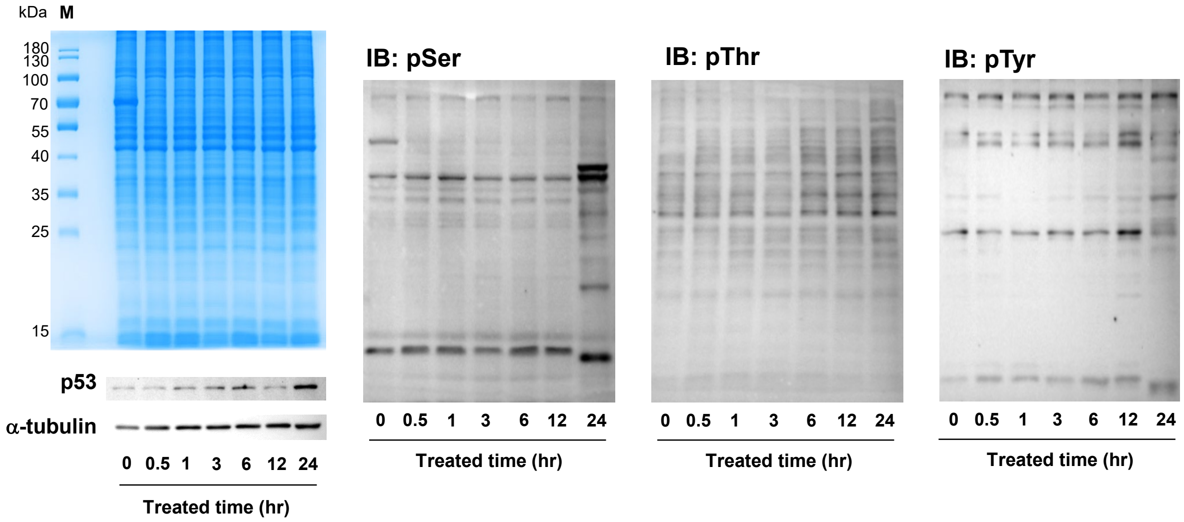

Figure S2. Monitoring of phosphorylation expression patterns by Western blots in Huh-7 cells after alpha–amanitin (α–AMA) treatment. Phosphorylation changes were observed up to 24 hr after treatment with 5 μM AMA in Huh–7 cells by Western blotting analysis. Phosphorylation patterns for the phospho–serine (pSer), phospho–threonine (pThr) and phospho–tyrosine (pTyr) were detected using corresponding antibodies.

Table S1. All identified phosphosites after treatment to α–AMA (5 μM) up to 12 hr to Huh–7 cells.

Identified as excel file

Table S2. Phosphorylation information classified in Spliceosome of KEGG category in Cluster 8

Proteins	Protein names	Gene	Position
S4R3H4	Apoptotic chromatin condensation inducer in the nucleus	ACIN1	S158, S780, S652, S330, S599,S328, S946
F8W1J5	Probable ATP-dependent RNA helicase DDX23	DDX23	S107, S109
J3KTA4	DEAD box protein 5	DDX5	S498
A2AB15	Pre-mRNA-splicing factor ATP-dependent RNA helicase DHX16	DHX16	S100<br>S47<br>S46<br>S43
K7END7	ATP-dependent RNA helicase DHX8	DHX8	H58
F8W6I7	Helix-destabilizing protein	HNRNPA1	S6
H7C1J8	Heterogeneous nuclear ribonucleoprotein A3	HNRNPA3	S92, S95
P61978	Heterogeneous nuclear ribonucleoprotein K	HNRNPK	S216, S284
Q15365	Poly(rC)-binding protein 1	PCBP1	S190, S173
A0A0A0MRN0	Pre-mRNA-splicing factor 38B	PRPF38B	S416, S418, S209
O75400	Pre-mRNA-processing factor 40 homolog A	PRPF40A	S883, S885, S888
Q5W011	Splicing factor 45	RBM17	S155
P49756	RNA-binding protein 25	RBM25	S677
Q9Y5S9	RNA-binding protein 8A	RBM8A	S46, S56
H3BUY5	RNA-binding motif protein, X chromosome	RBMX	S80, S198
O75533	Splicing factor 3B subunit 1	SF3B1	T248
E9PJ04	Splicing factor 3B subunit 2	SF3B2	S305, S307
O95391	Pre-mRNA-splicing factor SLU7	SLU7	S215
O75643	U5 small nuclear ribonucleoprotein 200 kDa helicase	SNRNP200	S225
P08621	U1 small nuclear ribonucleoprotein 70 kDa	SNRNP70	S226
G3V4X8	SNW domain-containing protein 1	SNW1	S62
J3KSR8	Serine/arginine-rich-splicing factor 1	SRSF1	S129, Y132, S94, S100
O75494	Serine/arginine-rich splicing factor 10	SRSF10	S156, S158, S160, S107, S133, S131, S129
Q01130	Serine/arginine-rich splicing factor 2	SRSF2	T25, S26, S189, S191
Q13243	Serine/arginine-rich splicing factor 5	SRSF5	S253, S250
Q13247	Serine/arginine-rich splicing factor 6	SRSF6	S303, S314, S316
Q13242	Serine/arginine-rich splicing factor 9	SRSF9	S211, S216, H213 Y221, Y214
Q13595	Transformer-2 protein homolog alpha	TRA2A	T202
P62995	Transformer-2 protein homolog beta	TRA2B	S85, Y86, S87
P26368	Splicing factor U2AF 65 kDa subunit	U2AF2	S79
H7C4V2	U2 snRNP-associated SURP motif-containing protein	U2SURP	T239
F8WC91	U4/U6.U5 tri-snRNP-associated protein 2	USP39	S82

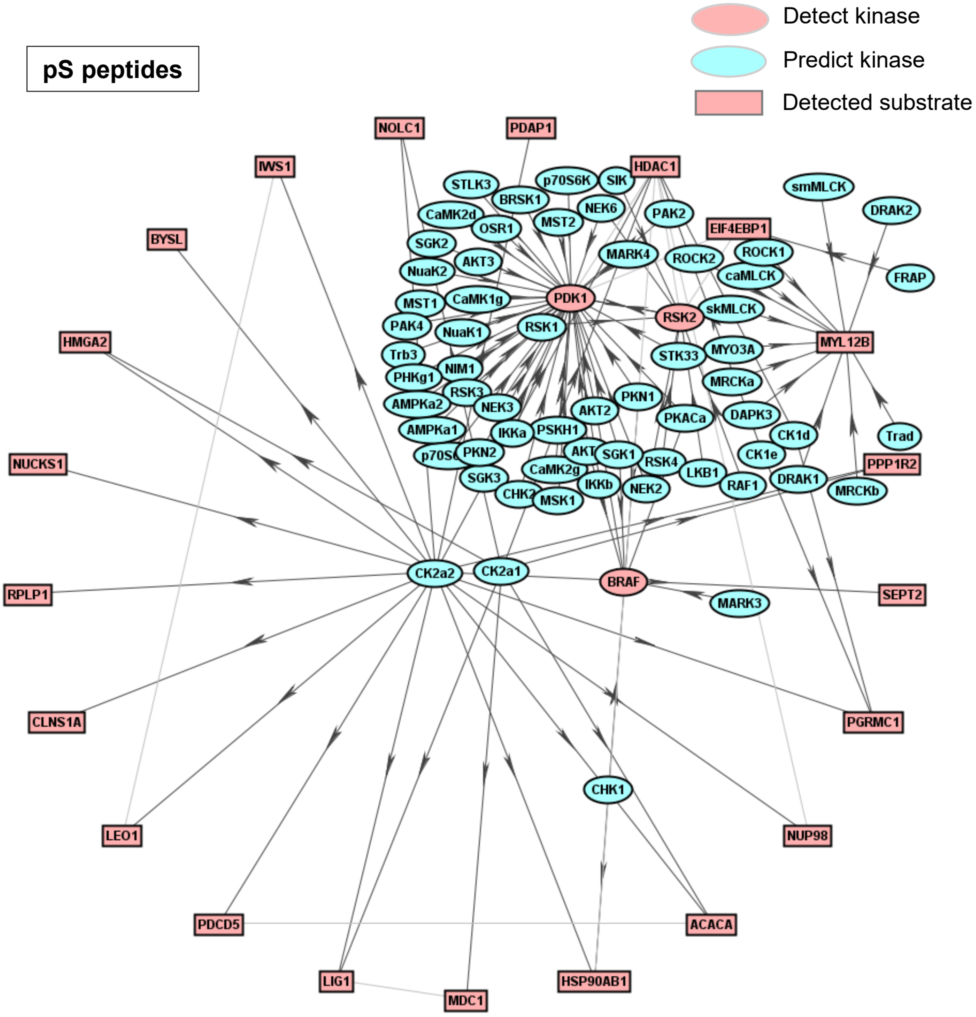

Figure S3. Kinase–substrate interaction analysis of phospho–serine proteins using iGPS 1.0. Circle (Kinase), Square (Substrate), Pink (Detected), Blue (Predicted).

Table S3. All identified phosphosites after treatment to α–AMA (10 μM) with ERK 1/2 inhibitor (5 μM) to Huh–7 cells

Identified as excel file

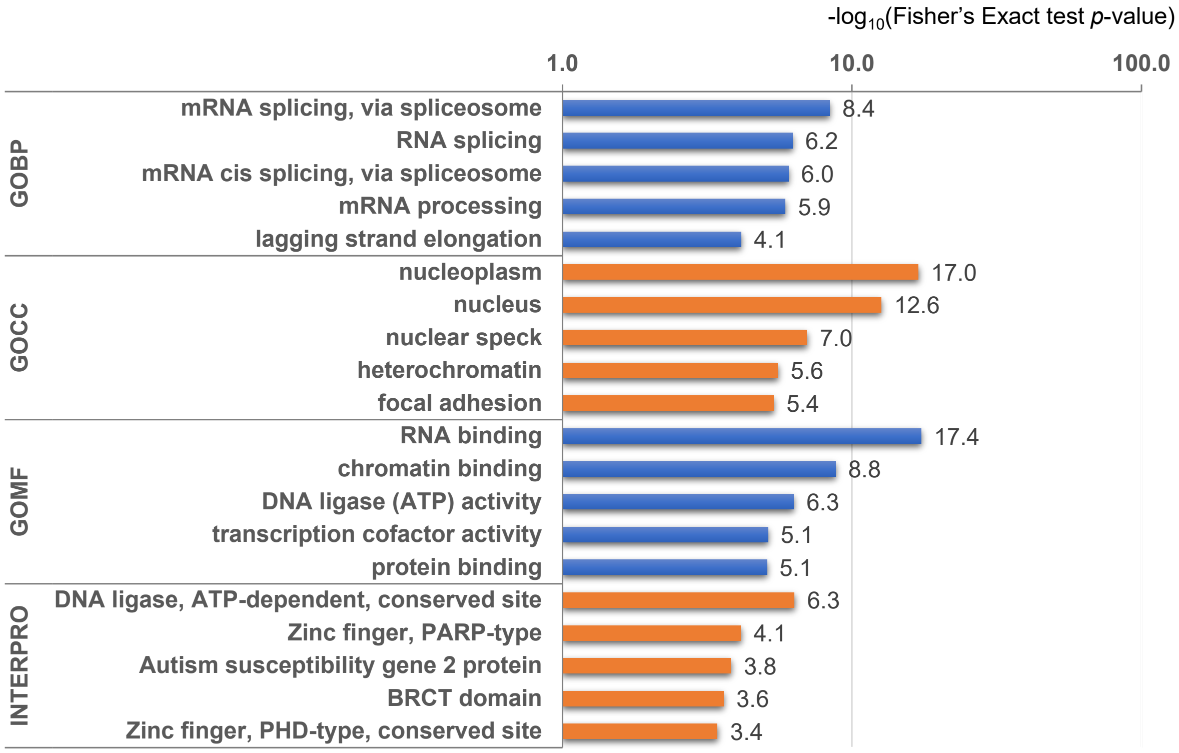

Figure S4. DAVID–generated Gene Ontology (GO) enrichment and Interpro of Cluster 5. Huh–7 cells were treated by α–AMA (10 μM) and/or ERK1/2 inhibitor (FR180204, 5 μM) for 24 hr.

Table S4. Phosphorylation information classified in Spliceosome of KEGG category in Cluster 5 after treatment to α-–AMA (10 μM) with ERK 1/2 inhibitor (5 μM) to Huh–7 cells

Protein ID	Name	Gene	Position
F8WC91	U4/U6.U5 tri-snRNP-associated protein 2	USP39	S154
Q5W011	Splicing factor 45 (Fragment)	RBM17	S212
S4R3H4	Apoptotic chromatin condensation inducer in the nucleus	ACIN1	S182, S185, S432
